# Correction: Cross-sectional survey evaluating the psychological impact of the COVID-19 vaccination campaign in patients with cancer: The VACCINATE study

**DOI:** 10.1371/journal.pone.0307924

**Published:** 2024-07-24

**Authors:** Daniela Tregnago, Alice Avancini, Lorenzo Belluomini, Ilaria Trestini, Marco Sposito, Jessica Insolda, Federica Bianchi, Teodoro Sava, Chiara Gaiani, Lidia Del Piccolo, Valentina Guarneri, Giuseppe Verlato, Ahmad Tfaily, Roberta Vesentini, Serena Zuliani, Sara Pilotto, Michele Milella

The eleventh author’s name is spelled incorrectly. The correct name is: Valentina Guarneri.
