# Supplementary figures and images for: Cross-sectional survey evaluating the psychological impact of the COVID-19 vaccination campaign in patients with cancer: The VACCINATE study
Source: PLoS One. 2024 Jan 25;19(1):e0290792. doi: 10.1371/journal.pone.0290792 (PMC10810487; doi:10.1371/journal.pone.0290792)

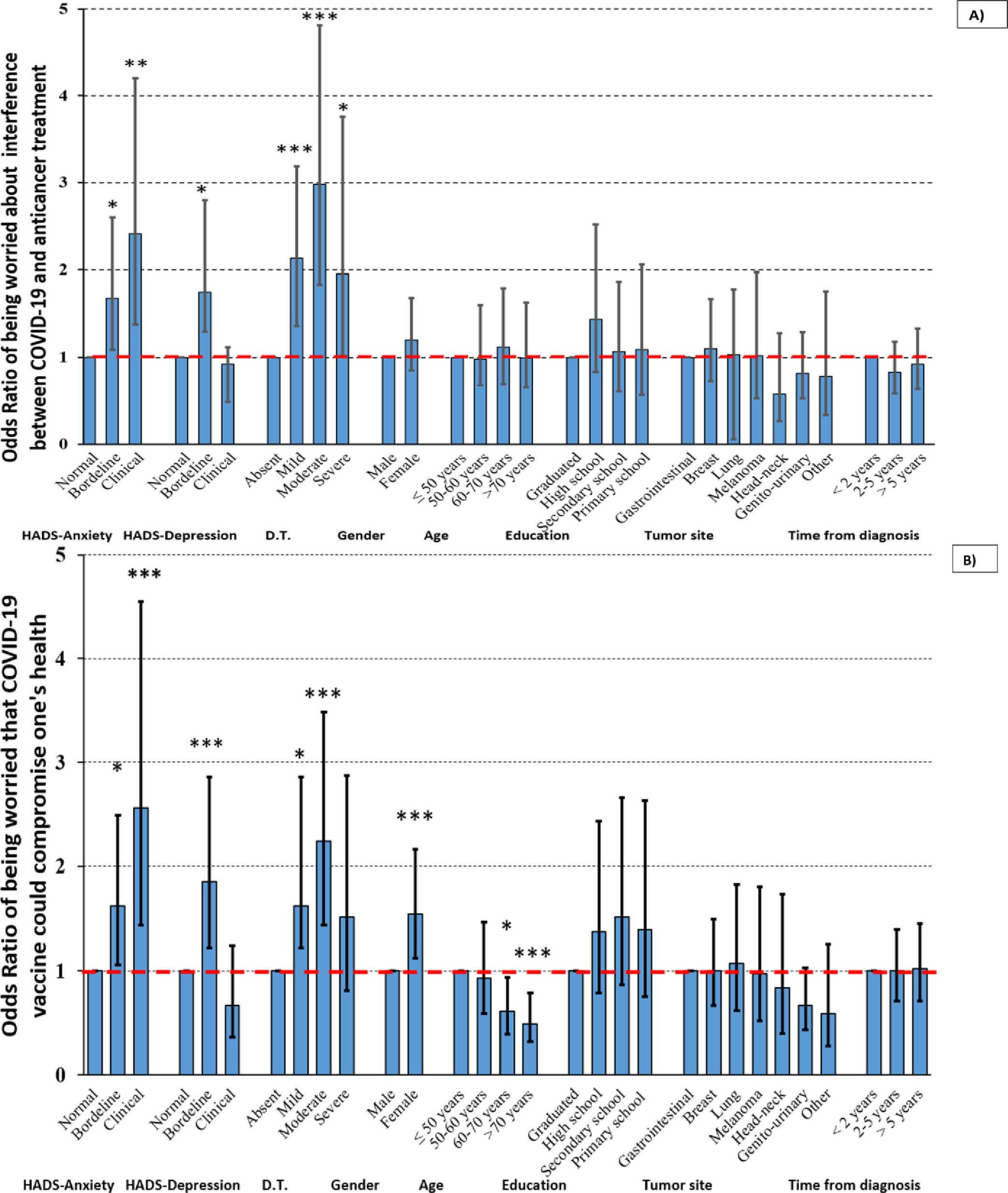

Supplement: S1 Fig — Odds Ratios (ORs) and 95% confidence intervals were derived by a multivariable logistic regression model. In the graph columns are ORs, bars are 95% confidence intervals. *p = 0.01; **p = 0.001; ***p<0.001. (TIF) [file pone.0290792.s001.tif]

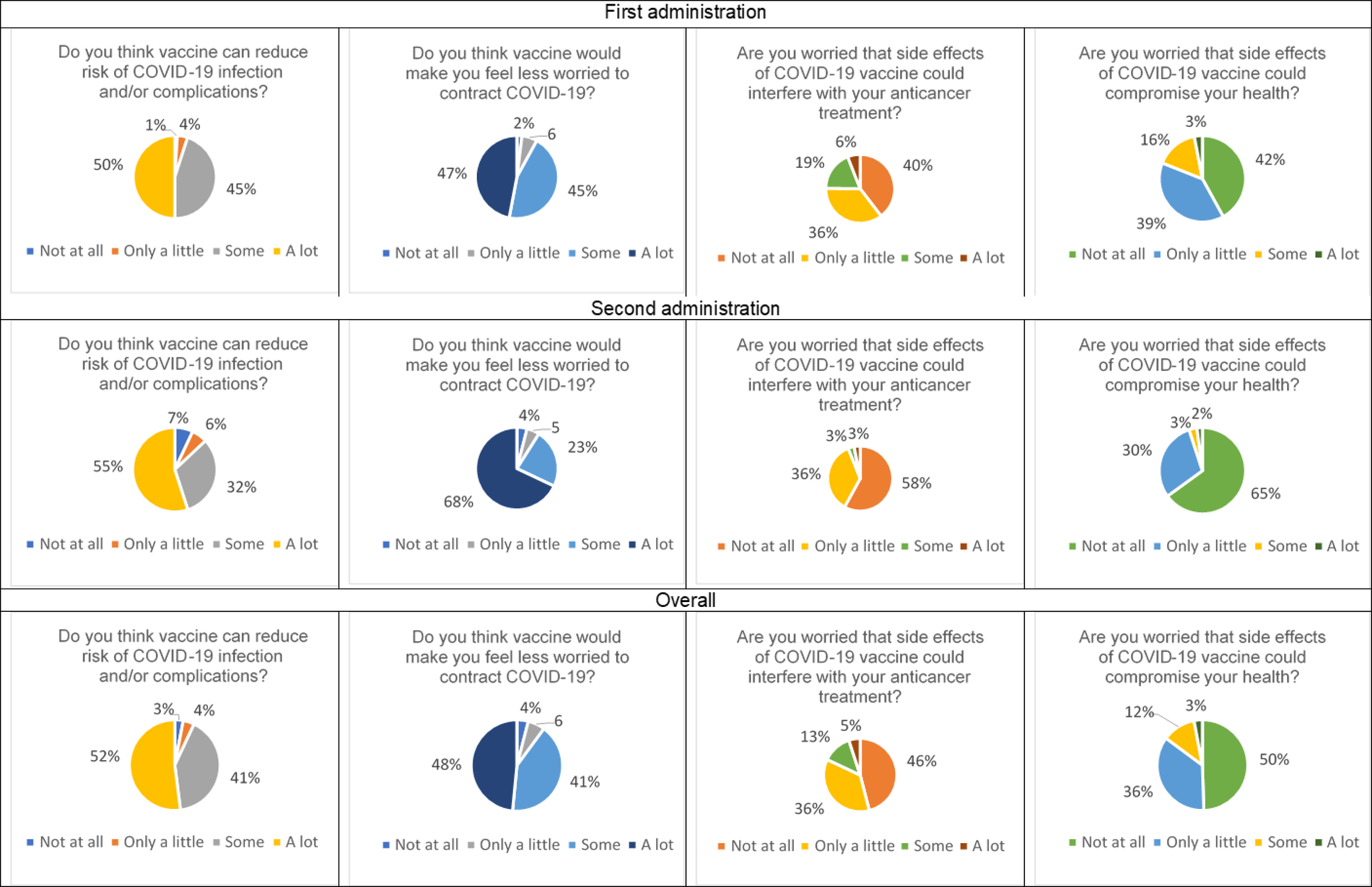

Supplement: S2 Fig — (TIF) [file pone.0290792.s002.tif]
