# Supplementary material for: Cross-sectional survey evaluating the psychological impact of the COVID-19 vaccination campaign in patients with cancer: The VACCINATE study
Source: PLoS One. 2024 Jan 25;19(1):e0290792. doi: 10.1371/journal.pone.0290792 (PMC10810487; doi:10.1371/journal.pone.0290792)
Supplement: S1 Table — (DOCX) [file pone.0290792.s003.docx]

| **HADS- Anxiety** | | | | |
| --- | --- | --- | --- | --- |
| **Tumor site** | *N (%)* | *N (%)* | *N (%)* |  |
|  | *Normal* | *Borderline* | *Clinical* | *p-value* |
| Gastro-intestinal | 271 (75.3) | 56 (15.6) | 33 (9.2) | .005 |
| Breast | 164 (65.3) | 43 (17.1) | 44 (17.5) |  |
| Lung | 76 (83.5) | 9 (9.9) | 6 (6.6) |  |
| Melanoma | 48 (78.7) | 7 (11.5) | 6 (9.8) |  |
| Head-neck | 35 (81.4) | 5 (11.6) | 3 (7.0) |  |
| Genito-urinary | 145 (81.5) | 23 (12.9) | 10 (5.6) |  |
| Rare | 18 (64.3) | 4 (14.3) | 6 (21.4) |  |
| Hematological | 5 (83.3) | 1 (16.7) | 0 (0) |  |
| Other | 8 (72.7) | 3 (27.3) | 0 (0) |  |
| **HADS- Depression** | | | | |

| **Tumor site** | *N (%)* | *N (%)* | *N (%)* |  |
| --- | --- | --- | --- | --- |
|  | *Normal* | *Borderline* | *Clinical* | *p-value* |
| Gastro-intestinal | 265 (74.7) | 61 (17.2) | 29 (8.2) | .107 |
| Breast | 188 (75.2) | 41 (16.4) | 21 (8.4) |  |
| Lung | 77 (88.5) | 6 (6.9) | 4 (4.6) |  |
| Melanoma | 53 (88.3) | 4 (6.7) | 3 (5) |  |
| Head-neck | 34 (79.1) | 5 (11.6) | 4 (9.3) |  |
| Genito-urinary | 143 (82.7) | 17 (9.8) | 13 (7.5) |  |
| Rare | 18 (66.7) | 5 (18.5) | 4 (14.8) |  |
| Hematological | 5 (83.3) | 1 (16.7) | 0 (0) |  |
| Other | 6 (54.6) | 3 (27.3) | 2 (18.2) |  |
