# Supplementary material for: Cross-sectional survey evaluating the psychological impact of the COVID-19 vaccination campaign in patients with cancer: The VACCINATE study
Source: PLoS One. 2024 Jan 25;19(1):e0290792. doi: 10.1371/journal.pone.0290792 (PMC10810487; doi:10.1371/journal.pone.0290792)
Supplement: S2 Table — *Spearman correlation. (DOCX) [file pone.0290792.s004.docx]

| **HADS-A** | **DT** **DISTRESS THERMOMETER** | | | | |
| --- | --- | --- | --- | --- | --- |
|  | *N (%)* | *N (%)* | *N (%)* | *N (%)* | *Correlation** |
|  | *Absent* | *Mild* | *Moderate* | *Severe* | rho = 0.4800  p <.000 |
| Normal | 200 (26) | 337 (44) | 201 (26) | 29 (4) |  |
| Borderline | 10 (7) | 34 (22) | 90 (60) | 17 (11) |  |
| Clinical  | 1 (0) | 6 (6) | 49 (47) | 47 (46) |  |
|  | **DT DISTRESS THERMOMETER** | | | | |

| **HADS-D** | *N (%)* | *N (%)* | *N (%)* | *N (%)* | *Correlation** |
| --- | --- | --- | --- | --- | --- |
|  | *Absent* | *Mild* | *Moderate* | *Severe* | rho = 0.3962  p <.000  |
| Normal | 200 (25) | 333 (42) | 215 (27) | 41 (5) |  |
| Borderline | 8 (6) | 28 (20) | 82 (58) | 22 (16) |  |
| Clinical | 3 (4) | 11 (14) | 35 (46) | 28 (36) |  |

*** Spearman correlation**
