# Supplementary material for: Cross-sectional survey evaluating the psychological impact of the COVID-19 vaccination campaign in patients with cancer: The VACCINATE study
Source: PLoS One. 2024 Jan 25;19(1):e0290792. doi: 10.1371/journal.pone.0290792 (PMC10810487; doi:10.1371/journal.pone.0290792)
Supplement: S3 Table — *Spearman correlation. (DOCX) [file pone.0290792.s005.docx]

| **HADS-Depression** | **HADS-Anxiety** | | | |
| --- | --- | --- | --- | --- |
|  | *N (%)* | *N (%)* | *N (%)* | *Correlation** |
|  | *Normal* | *Borderline* | *Clinical* | rho = 0.5142  p <.0001 |
| Normal | 703 (86) | 90 (11) | 25 (3) |  |
| Borderline | 69 (47) | 43 (29) | 35 (24) |  |
| Clinical | 14 (17) | 19 (24) | 47 (59) |  |

*** Spearman correlation**
