# Supplementary material for: Cross-sectional survey evaluating the psychological impact of the COVID-19 vaccination campaign in patients with cancer: The VACCINATE study
Source: PLoS One. 2024 Jan 25;19(1):e0290792. doi: 10.1371/journal.pone.0290792 (PMC10810487; doi:10.1371/journal.pone.0290792)
Supplement: S4 Table — (DOCX) [file pone.0290792.s006.docx]

| **Variable** | *Do you think vaccine can reduce the risk of COVID-19 infection and/or its complications?*  *(item 1)* | | *Do you think vaccine would make you feel less worried to contract COVID-19?*  *(item 2)* | | *Are you worried that COVID-19 side effects could interfere with your anticancer treatments?*  *(item3)* | | *Are you worried that side effects of COVID-19 vaccine could compromise your health?*  *(item4)* | |
| --- | --- | --- | --- | --- | --- | --- | --- | --- |
|  | **OR^a^ (95% CI)^b^** | **p-value^c^** | **OR^a^ (95% CI)^b^** | **p-value^c^** | **OR^a^ (95% CI)^b^** | **p-value^c^** | **OR^a^ (95% CI)^b^** | **p-value^c^** |
| *HADS-Anxiety* |  |  |  |  |  |  |  |  |
| Normal | 1 |  | 1 |  | 1 |  | 1 |  |
| Bordeline | 0.77 (0.29-2.00) | .588 | 0.86 (0.37-2.04) | .738 | 1.68 (1.09-2.60) | .017 | 1.63 (1.06-2.49) | .025 |
| Clinical | 1.20 (0.28-5.10) | .805 | 1.79 (0.46-6.90) | .401 | 2.41 (1.38-4.20) | .002 | 2.56 (1.44-4.55) | .001 |
| *HADS-Depression* |  |  |  |  |  |  |  |  |
| Normal | 1 |  | 1 |  | 1 |  | 1 |  |
| Bordeline | 0.52 (0.19-1.41) | .199 | 0.29 (0.12-0.70) | .006 | 1.75 (1.13-2.70) | .011 | 1.86 (1.22-2.86) | .004 |
| Clinical | 0.80 (0.16-3.95) | .789 | 0.54 (0.13-2.33) | .409 | 0.92 (0.49-1.70) | .784 | 0.67 (0.36-1.24) | .200 |
| *Distress Thermometer* |  |  |  |  |  |  |  |  |
| Absent | 1 |  | 1 |  | 1 |  | 1 |  |
| Mild | 0.71 (0.30-1.71) | .454 | 0.58 (0.26-1.30) | .187 | 2.13 (1.36-3.36) | .001 | 1.63 (1.22-2.86) | .019 |
| Moderate | 0.71 (0.29-1.74) | .449 | 0.56 (0.24-1.28) | .169 | 2.98 (1.84-4.81) | <.001 | 2.24 (1.44-3.48) | <.001 |
| Severe | 0.95 (0.20-4.49) | .950 | 0.68 (0.17-2.68) | .578 | 1.96 (1.02-3.76) | .043 | 1.52 (0.81-2.87) | .192 |
| *Sex* |  |  |  |  |  |  |  |  |
| Male | 1 |  | 1 |  | 1 |  | 1 |  |
| Female | 1.44 (0.67-3.09) | .344 | 2.04 (1.01-4.15) | .048 | 1.20 (0.85-1.68) | .298 | 1.55 (1.12-2.16) | .009 |
| *Age* |  |  |  |  |  |  |  |  |
| ≤ 50 years | 1 |  | 1 |  | 1 |  | 1 |  |
| 50-60 years | 1.13 (0.48-2.70) | .744 | 1.11 (0.49-2.51) | .808 | 0.98 (0.68-1.60) | .933 | 0.93 (0.59-1.47) | .766 |
| 60-70 years | 3.45 (1.42-8.38) | .006 | 2.86 (1.27-6.46) | .011 | 1.12 (0.69-1.79) | .652 | 0.61 (0.39-0.94) | .027 |
| >70 years | 4.11 (1.49-11.36) | .006 | 4.68 (1.84-11.88) | .001 | 0.99 (0.60-1.63) | .969 | 0.49 (0.32-0.79) | .004 |
| *Educational level* |  |  |  |  |  |  |  |  |
| Graduated | 1 |  | 1 |  | 1 |  | 1 |  |
| High school | 1.22 (0.30-4.92) | .783 | 0.94 (0.24-3.69) | .929 | 1.44 (0.83-2.52) | .197 | 1.38 (0.79-2.43) | .259 |
| Secondary school | 1.20 (0.30-4.77) | .793 | 0.81 (0.21-3.08) | .759 | 1.07 (0.61-1.87) | .815 | 1.52 (0.87-2.66) | .142 |
| Primary school or lower | 1.51 (0.33-6.95) | .599 | 0.69 (0.16-2.93) | .616 | 1.09 (0.57-2.06) | .797 | 1.40 (0.75-2.63) | .295 |
| *Cancer site* |  |  |  |  |  |  |  |  |
| Gastro-intestinal | 1 |  | 1 |  | 1 |  | 1 |  |
| Breast | 1.10 (0.43-2.80) | .838 | 0.76 (0.32-1.77) | .521 | 1.10 (0.73-1.67) | .653 | 1.00 (0.67-1.50) | .996 |
| Lung | 1.21 (0.32-4.61) | .781 | 1.59 (0.44-5.82) | .483 | 1.03 (0.060-1.78) | .905 | 1.07 (0.62-1.83) | .813 |
| Melanoma | 0.80 (0.23-2.83) | .729 | 0.65 (0.20-2.08) | .464 | 1.02 (0.53-1.97) | .948 | 0.97 (0.52-1.81) | .926 |
| Head-neck | 1.31 (0.29-5.99) | .727 | 0.79 (0.20-3.08) | .734 | 0.58 (0.27-1.28) | .180 | 0.84 (0.40-1.74) | .637 |
| Genito-urinary | 0.95 (0.38-2.42) | .919 | 0.92 (0.39-2.13) | .839 | 0.82 (0.53-1.29) | .397 | 0.67 (0.43-1.03) | .068 |
| Other | 0.25 (0.04-1.50) | .131 | 0.20 (0.04-1.14) | .070 | 0.78 (0.34-1.76) | .543 | 0.59 (0.28-1.26) | .173 |
| *Years of anticancer treatments* |  |  |  |  |  |  |  |  |
| < 2 years | 1 |  | 1 |  | 1 |  | 1 |  |
| 2-5 years | 2.15 (0.96-4.83) | .063 | 1.43 (0.72-2.84) | .307 | 0.83 (0.59-1.18) | .297 | 1.00 (0.71-1.40) | .994 |
| > 5 years | 0.82 (0.38-1.76) | .613 | 1.03 (0.50-2.12) | .935 | 0.92 (0.64-1.33) | .667 | 1.02 (0.71-1.46) | .909 |
| Note: ^a^OR (odds ratios), ^b^CI (confidence intervals), and ^c^ p-values from multivariable logistic regression model. | | | | | | | | |
