# Supplementary material for: Cross-sectional survey evaluating the psychological impact of the COVID-19 vaccination campaign in patients with cancer: The VACCINATE study
Source: PLoS One. 2024 Jan 25;19(1):e0290792. doi: 10.1371/journal.pone.0290792 (PMC10810487; doi:10.1371/journal.pone.0290792)
Supplement: S5 Table — (DOCX) [file pone.0290792.s007.docx]

| **Variable** | Total  (n=1089) | First administration (n=764) | Second administration  (n=325) | p-value |
| --- | --- | --- | --- | --- |
|  | *N (%)* | *N (%)* | *N (%)* |  |
| *HADS-Anxiety, score (0-21)^a^* |  |  |  | .828 |
| Normal | 217 (79.8) | 147 (79.0) | 70 (81.4) |  |
| Borderline | 39 (14.3) | 27 (14.5) | 12 (14.0) |  |
| Clinical | 16 (5.9) | 12 (6.5) | 4 (4.7) |  |
| *HADS-Depression, score(0-21)^a^* |  |  |  | .181 |
| Normal | 191 (71.8) | 129 (69.4) | 68 (79.1) |  |
| Borderline | 52 (19.5) | 41 (22.0) | 11 (12.8) |  |
| Clinical | 23 (8.6) | 16 (8.6) | 7 (8.1) |  |
| *Distress Thermometer,score (0-11)^b^* |  |  |  | .002 |
| Absent | 67 (27.3) | 34 (20.2) | 33 (42.8) |  |
| Mild | 81 (33.1) | 59 (35.1) | 22 (28.5) |  |
| Moderate | 74 (30.2) | 55 (32.7) | 19 (24.6) |  |
| Severe | 23 (9.4) | 20 (11.9) | 3 (3.8) |  |
