# Supplementary material for: Cross-sectional survey evaluating the psychological impact of the COVID-19 vaccination campaign in patients with cancer: The VACCINATE study
Source: PLoS One. 2024 Jan 25;19(1):e0290792. doi: 10.1371/journal.pone.0290792 (PMC10810487; doi:10.1371/journal.pone.0290792)
Supplement: S6 Table — (DOCX) [file pone.0290792.s008.docx]

| **HADS-A** | **ITEM 1:** *Do you think vaccine can reduce risk of COVID-19 infection and/or complications?* | | | | p-value |
| --- | --- | --- | --- | --- | --- |
|  | *N (%)* | *N (%)* | *N (%)* | *N (%)* |  |
|  | *Not at all* | *Only a little* | *Some* | *A lot* |  |
| Normal | 6 (3) | 10 (5) | 75 (37.5) | 109 (54.5) | .060 |
| Borderline | 0 (0) | 2 (5.4) | 23 (62.2) | 12 (32.4) |  |
| Clinical | 1 (6.3) | 0 (0) | 4 (25) | 11 (68.8) |  |
|  | **ITEM 2:** *Do you think vaccine would make you feel less worried to contract COVID-19?* | | | |  |
|  | *N (%)* | *N (%)* | *N (%)* | *N (%)* |  |
|  | *Not at all* | *Only a little* | *Some* | *A lot* |  |
| Normal | 11 (5.6) | 14 (7.1) | 78 (39.4) | 95 (48) | .176 |
| Borderline | 1 (2.7) | 3 (8.1) | 20 (54.1) | 13 (35.1) |  |
| Clinical | 0 (0) | 0 (0) | 4 (25) | 12 (75) |  |
|  | **ITEM 3:** *Are you worried that side effects of COVID-19 vaccine could interfere with your anticancer treatment?* | | | |  |
|  | *N (%)* | *N (%)* | *N (%)* | *N (%)* |  |
|  | *Not at all* | *Only a little* | *Some* | *A lot* |  |
| Normal | 96 (49.2) | 72 (37) | 20 (10.3) | 7 (3.6) | .005 |
| Borderline | 12 (33.3) | 13 (36.1) | 5 (13.9) | 6 (16.7) |  |
| Clinical | 6 (37.5) | 5 (31.5) | 5 (31.5) | 0 (0) |  |
|  | **ITEM 4:** *Are you worried that side effects of COVID-19 vaccine could compromise your health?* | | | |  |
|  | *N (%)* | *N (%)* | *N (%)* | *N (%)* |  |
|  | *Not at all* | *Only a little* | *Some* | *A lot* |  |
| Normal | 106 (53.8) | 70 (35.5) | 15 (7.6) | 6 (3.1) | .100 |
| Borderline | 12 (34.3) | 14 (40) | 8 (22.9) | 1 (2.9) |  |
| Clinical | 6 (37.5) | 7 (43.8) | 2 (12.5) | 1 (6.3) |  |
