# Supplementary material for: Cross-sectional survey evaluating the psychological impact of the COVID-19 vaccination campaign in patients with cancer: The VACCINATE study
Source: PLoS One. 2024 Jan 25;19(1):e0290792. doi: 10.1371/journal.pone.0290792 (PMC10810487; doi:10.1371/journal.pone.0290792)
Supplement: S7 Table — (DOCX) [file pone.0290792.s009.docx]

| **HADS-D** | **ITEM 1:** *Do you think vaccine can reduce risk of COVID-19 infection and/or complications?* | | | | *p-value* |
| --- | --- | --- | --- | --- | --- |
|  | *N (%)* | *N (%)* | *N (%)* | *N (%)* |  |
|  | *Not at all* | *Only a little* | *Some* | *A lot* |  |
| Normal | 3 (1.7) | 9 (5) | 64 (35.2) | 106 (58.2) | <.001 |
| Borderline | 1 (2) | 3 (6.1) | 29 (59.2) | 16 (32.7) |  |
| Clinical | 3 (13.6) | 0 (0) | 9 (40.9) | 10 (45.5) |  |
|  | **ITEM 2:** *Do you think vaccine would make you feel less worried to contract COVID-19?* | | | |  |
|  | *N (%)* | *N (%)* | *N (%)* | *N (%)* |  |
|  | *Not at all* | *Only a little* | *Some* | *A lot* |  |
| Normal | 9 (5) | 11 (6.1) | 68 (37.8) | 92 (51.1) | .311 |
| Borderline | 1 (2) | 3 (6.1) | 26 (53.1) | 19 (38.8) |  |
| Clinical | 2 (9.1) | 3 (13.6) | 8 (36.4) | 9 (40.9) |  |
|  | **ITEM 3:** *Are you worried that side effects of COVID-19 vaccine could interfere with your anticancer treatment?* | | | |  |
|  | *N (%)* | *N (%)* | *N (%)* | *N (%)* |  |
|  | *Not at all* | *Only a little* | *Some* | *A lot* |  |
| Normal | 89 (49.7) | 65 (36.3) | 19 (10.6) | 6 (3.4) | .236 |
| Borderline | 16 (34) | 18 (38.3) | 8 (17) | 5 (10.6) |  |
| Clinical | 9 (42.9) | 7 (33.3) | 3 (14.3) | 2 (9.5) |  |
|  | **ITEM 4:** *Are you worried that side effects of COVID-19 vaccine could compromise your health?* | | | |  |
|  | *N (%)* | *N (%)* | *N (%)* | *N (%)* |  |
|  | *Not at all* | *Only a little* | *Some* | *A lot* |  |
| Normal | 97 (53.9) | 68 (37.8) | 9 (5) | 6 (3.3) | <.001 |
| Borderline | 16 (34.8) | 18 (39.1) | 10 (21.7) | 2 (4.4) |  |
| Clinical | 11 (50) | 5 (22.7) | 6 (27.3) | 0 (0) |  |
