# Supplementary material for: Cross-sectional survey evaluating the psychological impact of the COVID-19 vaccination campaign in patients with cancer: The VACCINATE study
Source: PLoS One. 2024 Jan 25;19(1):e0290792. doi: 10.1371/journal.pone.0290792 (PMC10810487; doi:10.1371/journal.pone.0290792)
Supplement: Attachment — Submitted filename: Response to Reviewers_PLOSONE_PONE-D-23-06022_29.07.docx [file pone.0290792.s010.docx]

Verona, July 29^th^, 2023

Prof. Suzanne Rose, PhD

Academic Editor, PLOS ONE

Dear Editor,

Enclosed please find a thoroughly revised version of the manuscript entitled ***“Cross-sectional survey evaluating the psychological impact of the COVID-19 vaccination campaign in patients with cancer: The VACCINATE study"* [PONE-D-23-06022]** that we wish to resubmit for publication in Plos One*.*

We would like to thank the reviewers for the thoughtful and stimulating comments that have prompted us to update and clarify several points and revise the manuscript accordingly.

We thus hope that the quality of the manuscript has now substantially improved, so that it may be reconsidered for publication.

A point-by-point rebuttal description of the performed revisions follows herein:

**Editor**

|  | **Editor’ Comments:** | **Response:** |
| --- | --- | --- |
| **1)** | PLOS ONE's style requirements | Done |
| **2)** | State what role the funders took in the study | The following comment was added to the financial disclosure statement. *The funders had no role in study design, data collection and analysis, decision to publish, or preparation of the manuscript.* |
| **3)** | ORCID iD for the corresponding author | The link to automatically link the OrcidID to the submission does not work properly; the OrcidID for the corresponding author (prof. Michele Milella) is: 0000-0002-3826-5237 |
| **4)** | We note that you have included the phrase “data not shown” in your manuscript. Unfortunately, this does not meet our data sharing requirements. PLOS does not permit references to inaccessible data. | Data not shown in the previous version of the manuscript have now been provided as Supplementary Tables S1 |
| **5)** | Include captions for your Supporting Information files at the end of your manuscript, and update any in-text citations to match accordingly | Done |

**Referee #1**

|  | **Reviewer's Comments:** | **Response:** |
| --- | --- | --- |
|  | General comments. | We thank the reviewer for acknowledging the potential interest of our piece of work and for giving us important suggestions on how to improve the manuscript. |
| **1)** | The article is reasonably well organized and written. While quite well-written in English, throughout the paper there are some linguistic inaccuracies and imprecision in language that makes the meaning unclear (esp. in  the methods section). Editing for clarity of communication is needed. | We thank the reviewer for his/her suggestion. Language editing has been implemented throughout the manuscript. |
| **2)** | The title is misleading since the term “prospective, cross-sectional study” is  contradictory. | According to the reviewer's suggestion, we have revised the title into: ***Cross-sectional survey evaluating the psychological impact of the COVID-19 vaccination campaign in patients with cancer: The VACCINATE study*** |
| **3)** | Regarding Abstract/Background: Please name the term (patients) in full first before abbreviating it. The term "psychological status" is somewhat inaccurate and should be replaced with a more precise term that better describes the constructs being measured. Regarding Abstract/Methods and findings: Please add some information on patient sample size, response rate and sociodemographic characteristics like median age and sex. | We have thoroughly revised the abstract, according to the reviewer's suggestions. |
| **4)** | Regarding Introduction: The authors occasionally speak of “a substantial body of evidence” or “a growing body of evidence” (p.3., ll. 72/92) but refer only to a single citation, which does not really support the claim thus made. | According to the reviewer's suggestion, we have rephrased the relative paragraphs of the Introduction and added updated relevant references. |
| **5)** | The term "active cancer patient" (p.3, l. 99) is somehow misleading. "Patients with active cancer" does seem to be more appropriate | The term "patients with active cancer" was substituted for "active cancer patients" throughout the manuscript, according to the reviewer's suggestion. |
| **6)** | The authors should clarify the objective of the study, as it is not sufficiently compelling to state that "results will be reported" without stating the specific intent.  I am not sure what "patient characteristics" means. The term should be described in more detail, i.e., whether it is sociodemographic and clinical characteristics | According to the reviewer's suggestion, we have rephrased the paragraph of the Introduction regarding study aims. |
| **7)** | The authors stated that they have assessed patients who refused the vaccination. Were additional variables collected describing them in terms of age, sex, tumor-specific variables to compare them to patients who received the vaccination?  Please move the sentence "the number of patients who refused vaccination was calculated" to the statistical analyses section (p. 4, line 112). | We thank the reviewer for his/her comment. The study was designed in such a way that accepting the vaccination proposal and signing the informed consent form were the only mandatory inclusion criteria; this, unfortunately, precluded the possibility to collect information on patients refusing vaccination.  The sentence on the number of patients who refused vaccination has been moved to statistical analyses section as suggested.  Data were collected in the context of the COVID-19 vaccination campaign promoted by ROV between March and May 2021, which was directed to adult (≥18 years) patients with active cancer, defined as: patients with a new cancer diagnosis, scheduled to receive any systemic anticancer treatment; patients on ongoing systemic cancer treatment or who had completed systemic treatment within 6 months from the vaccination proposal. The only mandatory inclusion criteria to participate in the VACCINATE study were: 1) accepting the vaccination proposal, and 2) signing the study-specific informed consent form.  Eligibility criteria for both the vaccination campaign and the VACCINATE study have now been clarified in the *Methods* section of the revised manuscript (p.5, ll. 88/89). |
| **8)** | The authors should specify the eligibility criteria ii) confirmed diagnosis of cancer, since the term “active cancer” was previously introduced. Where there any exclusion criteria in terms of treatment, cancer site, stage, etc.? |  |
| **9)** | Regarding Methods, Questionnaires: Please provide the appropriate references for the questionnaires used.  Is there a rationale for why the metric scale of the DT was structured categorically? Research support a cut-off score of 5 on the DT to indicate patients with clinically elevated distress levels.  I don't quite understand how the item "Do you think the vaccine makes you less afraid of contracting COVID-19?" is supposed to represent an interference with cancer treatments. | We thank the reviewer for his/her suggestions.  We have now appropriately referenced the questionnaires utilized.  Concerning the metric scale of the DT, as cutoff scores for specific patient populations may vary, we elected to analyze DT categorically, based on the work of Mitchell and coll. [ref. 24]. This has now been clearly stated in the *Methods* section of the revised manuscript (p.5-6, ll. 105/107).  Item 2 has been developed as complementary to Item 1, in that Item 1 asks whether the subject *rationally* believes that vaccination could reduce the risk of contracting COVID-19 and Item 2 asks whether this would make him/her *less afraid*. Both items reflect the subject's confidence that vaccination would avoid contracting COVID-19 and therefore not being able to access the hospital for anticancer treatment. This has been more clearly explained in the *Methods* section of the revised manuscript (p.6, ll. 108/112). |
| **10)** | Regarding Methods, Statistical analysis: Please change the term “categorical variables” to “continuous variables” (p.5, l.150). Item number 3 mentioned in the statistical analysis for the primary outcome does not match with the item number from the description of study measures (where “interferences with anticancer treatments” refers to item number 2). It is unclear what the authors meant by “significant interference” (p.5, l.158) – please clarify.  Some expressions used to describe the statistical analyses seem inappropriate, i.e. “main determinants”.  This section contains the objectives of the study, which should be mentioned in the last paragraph of the introduction. This section needs to be revised for a detailed description of all statistical analyses used to test the research questions.  The authors considered two independent samples (first and second vaccination) as one sample (p.5, ll.163-66). It is unclear what the authors mean by this, as the following results are based on comparisons between the two groups. | According to the reviewer's suggestions, we have extensively revised *Statistical analysis* section of the Methods, eliminating imprecisions and correcting statistical terms, where appropriate.  We have also more clearly explained the rationale for treating the first- and second-dose populations as either two separate cohorts or a single sample (p.6, ll. 125/127). |
| **11)** | Regarding results, patient sample: p.5-6, ll. 174-78 This sentence needs rephrasing. It is not clear what the authors mean by “significant imbalances”. Please clarify. | The sentence has been rephrased according to the reviewer's suggestion (p.7, ll. 136/137) of the revised manuscript). |
| **12)** | Regarding results, table 1 and other tables including p values: Please do not include the zero before the decimal point reporting the p values. | We have corrected the tables accordingly, if this is compliant with journal style. |
| **13)** | Regarding results, suppl. Table S3: The abbreviation of the distress thermometer must be DT, not TD. Please rectify. The decimals points of the p value amounts to three not four as displayed in table S3 | We have corrected Supplementary Table S3 according to the reviewer's comments. |
| **14)** | Regarding results, Figure 2: It was a little difficult to capture the diagrams. Consider a similar color scheme for similar items to make visual comparisons easier | We have revised Figure 2 according to the reviewer's comments. |
| **15)** | Regarding results, logistic regression analysis: The heading does not summarize the content of the section below it. It is not clear how the variables are related to each other (direction of the correlation). To me, it is confusing that response frequencies are reported within the results of the regression analysis. This section needs restructuring. | We thank the reviewer for his/her extremely useful comment; following his/her advice, we have completely re-written the paragraph (page 10, lines 165-176 of the revised manuscript), provided additional Figures (Figure 3A-B and Supplementary Figure S1A-B of the revised manuscript), moved former Table 3 in the Supplementary materials (Supplementary Table S4 of the revised manuscript), and eliminated several Supplementary tables (former Supplementary Tables S4, S5, S6). We believe these changes will contribute to clarifying the nature and meaning of the data being presented and the overall readability of the manuscript. |
| **16)** | Regarding results, comparison of hospitals: It would certainly be interesting to describe the patient characteristics of the second sample from Camposampiero Hospital | We completely agree with the reviewer's comment; unfortunately, it was not possible to retrieve the socio-demographic characteristics of patients vaccinated at the Camposampiero Hospital. |
| **17)** | Regarding discussion: I’m not sure what the term “overall figures” means. Please clarify.  Please remove the references for tables in the discussion. | According to the reviewer's suggestion, we have thoroughly revised the Discussion section, highlighting strengths and limitations, our interpretation of the results, implications for practice, and further research needs in the final paragraph (p.12-13, ll. 201/254) of the revised manuscript). |
| **18)** | Regarding discussion: Unfortunately, a detailed description of the nature and significance of the results and their implications is lacking. The authors should better elaborate the implications for practice and further research needs. |  |

**Referee #2**

|  | **Reviewer's Comments:** | **Response:** |
| --- | --- | --- |
| **1)** | It is not clear the meaning of "Prospective..." in the title given that the study design is cross-sectional. I suggest to delete it from the title. | According to the reviewer's suggestion, we have revised the title into: ***Cross-sectional survey evaluating the psychological impact of the COVID-19 vaccination campaign in patients with cancer: The VACCINATE study*** |
| **2)** | Number of enrolled patients: if I correctly understood, out of 1794 patients invited for vaccination, 1763 accepted vaccination and 1089 accepted to take part in the study-i.e., a participation rate of 61.8%. What are the characteristics of vaccinated patients that refuse study participation? The authors should comment on this potential selection bias. | We thank the reviewer for his/her comment. The study was designed in such a way that accepting the vaccination proposal and signing the informed consent form were mandatory inclusion criteria; this, unfortunately, precluded the possibility to collect information on patients refusing vaccination or patients accepting vaccination, but not agreeing to participate in the study; such limitation is now clearly acknowledged in the Discussion section (p. 13, ll. 237/240 of the revised manuscript). |
| **3)** | The authors made hundreds of statistical tests: a comment on the role of multiple testing before acceptance of statistical significant differences would be welcomed | We thank the reviewer for his/her suggestion. Adjusting for multiple testing bias is mandatory in confirmatory analyses, such as randomized controlled trials, but not in exploratory analyses, such as observational exploratory studies. Anyway, to cope with this problem we identified primary endpoints (items 3 and 4), and secondary endpoints (items 1 and 2), and we particularly focused on the former. Moreover, we performed both univariable analyses where single variables are tested one by one, and multivariable analysis, where the most relevant variables are evaluated altoghether by ordered logistic regression models. Hence, the risk of possible multiple testing bias, which could affect univariable analysis, was dealt with in the final multivariable analysis. For all the above-mentioned reasons, we do not think that the use of multiplicity adjustment, such as Šidák’s or Bonferroni’s corrections, would significantly improve the present manuscript, but are willing to implement them, should the Editor deem it appropriate. |

Moreover, we have checked carefully the author checklist in order to provide all the requested manuscript details.

We look forward to hearing from you regarding our submission and hope that the manuscript is now acceptable for publication in your prestigious Journal and of interest to PLOS One readers. We would be glad to respond to any further questions and comments that you may have.

Best regards,

Michele Milella, Sara Pilotto, and Daniela Tregnago on behalf of all authors.
